# Gut *Streptococcus* is a microbial marker for the occurrence and liver metastasis of pancreatic cancer

**DOI:** 10.3389/fmicb.2023.1184869

**Published:** 2023-06-14

**Authors:** Jinru Yang, Yuxi Ma, Qiaoyun Tan, Bin Zhou, Dandan Yu, Min Jin, Tao Zhang, Junli Liu, Hongli Liu

**Affiliations:** ^1^Cancer Center, Union Hospital, Tongji Medical College, Huazhong University of Science and Technology, Wuhan, China; ^2^Institute of Radiation Oncology, Union Hospital, Tongji Medical College, Huazhong University of Science and Technology, Wuhan, China

**Keywords:** *Streptococcus*, pancreatic cancer, liver metastasis, gut microbiome, 16S rRNA sequencing

## Abstract

**Background:**

Gut microbiome plays an indispensable role in the occurrence and progression in various diseases. The incidence of pancreatic cancer (PC) and liver metastasis (PCLM) are high, most of them are found in advanced stage. Therefore, it is particularly necessary to search for predictive biomarkers, which are helpful for early detection and treatment, and thus improve the survival rate and quality of life of PC patients.

**Methods:**

We retrospectively analyzed 44 pancreatic cancer patients (P group, *n* = 44) and 50 healthy people (N group, *n* = 50) from March 21, 2021 and August 2, 2022. Among all PC patients, we divided them into liver metastasis group (LM group, *n* = 27) and non-liver metastasis group (non-LM group, *n* = 17). DNA was extracted and 16S ribosomal RNA (16S rRNA) gene sequencing was performed. SPSS was used for statistical analyses and all bioinformatics analyses were based on QIIME2, *p* < 0.05 were considered statistically significant.

**Results:**

The microbial richness and diversity of group P and LM were higher than that of group N and non-LM. LEfSe analysis found that *Streptococcus* was a significantly different microorganism, which was further identified by random forest (RF) model, and its ability to predict PC and PCLM was verified by ROC curve.

**Conclusion:**

We demonstrated significant differences in intestinal microbiome composition between PC patients and healthy people, and found that *Streptococcus* is a potential biomarker for early prediction of PC and PCLM, which is critical for early diagnosis of diseases.

## Introduction

According to the GLOBOCAN’s statistics in 2020 (Sung et al., 2021), there were approximately half million new cases of pancreatic cancer (PC) worldwide (2.6%/19.3 million), however, its mortality rate accounts for 4.7% of all cancer specific deaths. Although in recent years, with the rise of new treatment methods including immunotherapy and targeted therapy, as well as the deepening understanding of complex mechanisms, the overall 5-year survival rate of PC has not changed much. It is estimated that PC may become one of the main causes of cancer-related deaths in the future (Neoptolemos et al., 2018). The pancreas is a retroperitoneal organ, due to its concealed location, is surrounded by the duodenum, which affects its observation (Zhang et al., 2018), and lacks special symptoms and develops rapidly. Thus, PC is often found in the late stage. Liver metastasis (LM) is one of the most common modes of PC metastasis, and the main cause of treatment failure and death in advanced PC patients (Zheng et al., 2020). At present, there is no effective prediction method for PC. In clinical practice, traditional methods such as serum tumor markers, imaging examination or biopsy are commonly used to screen and diagnose PC. However, when the indicators change, the disease has progressed (Zhou et al., 2017). Therefore, it is very necessary to find convenient, non-invasive and inexpensive PC prediction biomarkers, which will help in the early detection and treatment of PC.

Pancreatic carcinogenesis is related to a variety of risk factors, including genetic factors, inflammatory factors, and stimulating factors, such as smoking, drinking, etc. Currently, researchers have focused on intestinal microorganisms (Klein, 2021). The human intestinal microbes and its metabolites constitute a complex microecology. There are approximately 10^14^ types of bacteria in the human digestive tract, mainly distributed in the colon and rectum (Sender et al., 2016). It plays a crucial role in many life processes, such as promoting metabolism, regulating energy storage, activating immune system, and maintaining intestinal homeostasis (Deschasaux et al., 2018; Hartmann et al., 2019). Under normal circumstances, bacteria in the digestive tract maintain a relative balance of species and quantity through symbiosis, competition and antagonism, and maintain dynamic balance with the host. Once the balance is abnormal or disrupted, it can cause bacteria disorders and lead to a series of diseases (Fu et al., 2022). More importantly, the toxic products of intestinal microorganisms have been identified as possible carcinogens, such as improving the tumorigenic effect by triggering double stranded DNA damage. At the same time, intestinal microorganisms are associated with a variety of risk factors related to pancreatic cancer, such as diabetes, chronic pancreatitis and obesity, which may potentially affect PC and PCLM. Based on this background, this study took pancreatic cancer as the starting point to explore the functional mechanism of intestinal microorganisms in liver metastasis, hoping to find biomarkers that predict PC and PCLM, and provide theoretical basis for prolonging the survival of PC patients.

## Methods

### Patients

We retrospectively analyzed 44 untreated PC patients (pancreatic cancer group, P group) and 50 matched healthy volunteers (Normal group, N group) between March 21, 2021 to August 2, 2022. All patients were selected through preset inclusion and exclusion criteria, the inclusion criteria were as follows: (1) all cases were confirmed as pancreatic cancer at the cancer center of Union Hospital, Tongji Medical College, Huazhong University of Science and Technology; (2) the patients had not received anti-tumor treatment, except for surgery, and stool samples were collected 3 weeks after the surgery; (3) no antibiotics or other drugs that may affect the intestinal flora were taken before the samples collection; (4) no intestinal invasive operations, such as gastrointestinal endoscopy and enema were performed; (5) without bile duct obstruction; and (6) the patients knew the contents of the study and had signed the informed consent. Patients who do not meet the inclusion criteria will be excluded. Among the PC patients, we divided them into liver metastasis group (LM group, *n* = 27) and non-liver metastasis (non-LM group, *n* = 17) to find the key intestinal microorganisms and related metabolic pathways that distinguish PC and PCLM.

For all PC patients, baseline clinical-pathological characteristics, including age, gender, body mass index (BMI), tumor sites, pathological types, whether metastasis, metastasis sites, whether surgery, lines of treatment, Eastern Cooperative Oncology Group (ECOG) performance and baseline bilirubin value were available for review. This retrospective study was approved by the Ethical Committees of Union Hospital, Tongji Medical College, Huazhong University of Science and Technology (No. 2014-041).

### Sample collection and 16S rRNA sequencing

Fecal samples of the PC patients were collected by the trained medical staff before anti-tumor treatment, put each sample in a 50 mL sterile specimen collection box, then immediately transfer to a −80°C refrigerator for storage. After all samples were collected, they were sent for examination. The samples’ total microbiome DNA was isolated with Omega Mag-Bind Soil DNA kit (Omega Bio-Tek, Norcross, GA, United States), and the concentration and purity of the DNA were determined (Yu et al., 2021). The V3-V4 variable regions of the qualified samples were sequenced using Illumina platform (Illumina, San Diego, CA, United States) and the original data were filtered by the dada2 method of Quantitative Insights into Microbial Ecology2 (QIIME2) software (v2019.4) (Rai et al., 2019), and the effective data were stored in FASTQ format. On this basis, the similarity sequences were clustered as amplicon sequence variants (ASVs), then the Naive Bayes classifier in QIIME2 software was used to cross compare with the Greengenes database (release 13.8)^1^ (DeSantis et al., 2006) for species annotation.

### Statistical and bioinformatic analyses

SPSS software (version 22.0, SPSS Inc., Chicago, IL, United States) were used for statistical analysis. Graphpad prism 8.0 (GraphPad Software Inc., San Diego, CA, United States) was used to draw charts. The patient characteristics were compared by Chi-squared test and Student’s *t*-test. All tests were performed by two-sided, and *p* < 0.05 were considered statistically significant. The 16S rRNA sequencing data were analyzed based on QIIME2 software. Principal coordinates analysis (PCoA) (Shi et al., 2020), Linear discriminant analysis (LDA) and effect size (LEfSe) (Segata et al., 2011), Kruskal-Wallis test, permutational multivariate analysis of variance (PERMANOVA), random forest (RF) model (Liu et al., 2020) and other bioinformatic methods were used to perform hypothesis test of intergroup diversity and predict the microbial metabolism function.

## Results

### Demographic characteristics

A total of 44 GC patients between March 21, 2021 and August 2, 2022 were included in our retrospective study. There was no statistical difference in baseline information between the two groups, the baseline characteristics of 44 PC patients were summarized in Table 1. In the PC population, 61.36% were under 65 years old, and there were 23 (52.27%) females and 21 (47.73%) males. The body mass index (BMI) was 22.43 ± 2.75 kg/cm^2^. The tumors mainly located in the whole (40.91%) and head (40.91%) of the pancreas. The majority pathological classification was adenocarcinoma, accounting for 81.82%. Thirty-two (72.73%) PC patients suffered from metastasis, including twenty-seven (27/32, 84.38%) liver metastasis (LM), eighteen (18/32, 56.25%) distant lymph node metastasis, nine (9/32, 28.13%) peritoneal metastasis and four (4/32, 12.50%) lung metastasis, only one quarter patients underwent surgery before treatment.

**Table 1:** Demographic characteristics of PC patients (*n* = 44).

Variable	*n* (%)
Age (y)	
<65	27 (61.36%)
≥65	17 (38.64%)
Gender	
Male	21 (47.73%)
Female	23 (52.27%)
BMI	22.43 ± 2.75
Tumor sites	
Head	18 (40.91%)
Body	3 (6.82%)
Tail	5 (11.36%)
Whole	18 (40.91%)
Pathological types	
Adenocarcinoma	36 (81.82%)
Squamous cell carcinoma	3 (6.82%)
Other	5 (11.36%)
Whether metastasis	
Yes	32 (72.73%)
No	12 (27.27%)
Metastasis sites (*n* = 32)	
Liver	27 (84.38%)
Lung	4 (12.50%)
Distant lymph node	18 (56.25%)
Peritoneum	9 (28.13%)
ECOG performance	
0	43 (97.73%)
1	1 (2.27%)
Whether surgery	
Yes	11 (25.00%)
No	33 (75.00%)
Total bilirubin (TBIL) (μmol/L)	15.90 ± 12.57
Direct bilirubin (DBIL) (μmol/L)	5.63 ± 4.83
Indirect bilirubin (IBIL) (μmol/L)	11.86 ± 12.39

### 16S rRNA sequencing results

#### Sequencing data processing

**Figure 1:**
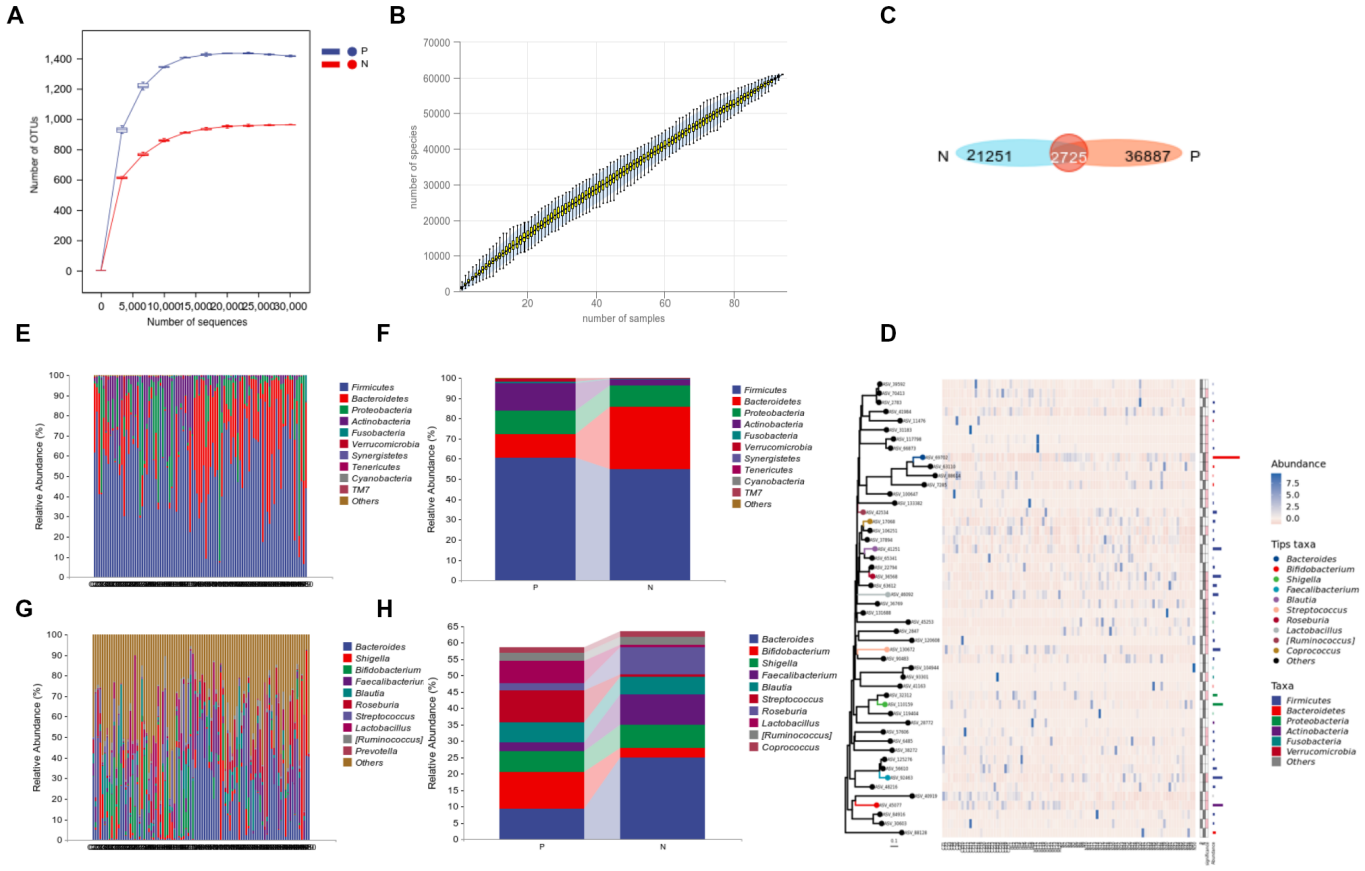
16S rRNA sequencing data processing and species composition of P and N groups. **(A)** Refraction curve; **(B)** species accumulation curve; **(C)** Venn gram of ASV/OTUs; **(D)** phylogenetic tree plot; **(E–H)** compositional analysis of each sample **(E,G)** and group **(F,H)** at the phylum level **(E,F)**, and at the genus level **(G,H)**.

The rarefaction curve and species accumulation curve indicated that the sample sequencing depth was sufficient and the annotated species were rich, with more than 6*10^4^ species, which can better represent the microbial flora information of each group, as shown in Figures 1A,B. Venn diagram showed that 36886 and 21251 ASV/OTUs were clustered in P group and N group, respectively, (Figure 1C).

#### Microbial diversity analysis

**Figure 2:**
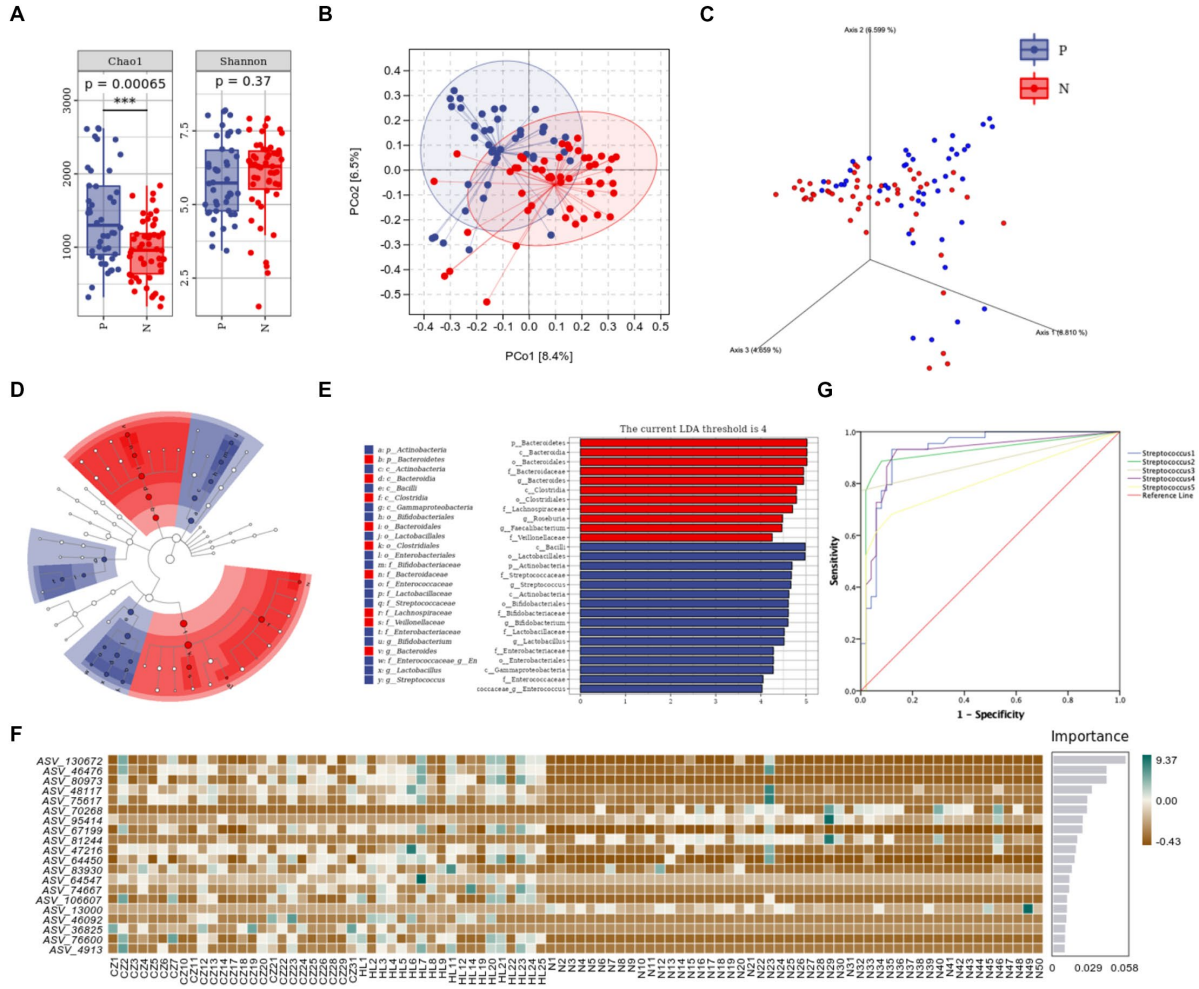
Species diversity results of P group and N group. **(A)** Alpha diversity; **(B)** PCoA of beta diversity (*R*^2^ = 4.477, *p* = 0.001); **(C)** 3D-PCoA; **(D)** taxonomic branch diagram of LEfSe (LDA threshold = 4); **(E)** LDA histogram; **(F)** RF model; **(G)** ROC curves. ***p<0.001.

For alpha diversity, Chao1 and Shannon indexes were used to represent the gene richness and number, both of them were higher in the P group than in the N group (*p* < 0.001, *p* = 0.37) (Figure 2A). According to Bray-Curtis distance algorithm, the graphs of principal coordinate analysis (PCoA) and 3D-PCoA^2^ were drawn to display beta diversity, β-diversity between P and N group was large (*R*^2^ = 4.477, *p* = 0.001), indicating that there were composition differences between two groups (Figures 2B,C). Axis 1, 2, and 3 of 3D-PCoA explain 8.810, 6.599, and 4.659% of variance, respectively.

#### Compositional analysis between PM and non-PM groups

According to the relative abundance (Figures 1E,G), intragroup cumulative abundance (Figures 1F,H) and phylogenetic tree plot (Figure 1D) of the top 10 species in each sample. At phylum level, *Firmicutes, Bacteroidetes, Proteobacteria* and *Actinobacteria* in both groups rank in the top four (Figure 1D). Among them, the relative abundance of *Verrucomicrobia* in P group is 9.89% higher than that in N group (Figures 1E,F), and at the genus level, the relative abundance of *Streptococcus, Lactobacillus*, and *Bifidobacterium* in the P group was significantly increased, especially *Streptococcus*, which increased by 9.04 times in the N group (Figures 1G,H).

Linear discriminant analysis (LDA) was conducted to estimate the effect size (LEfSe) of each differential flora. The LDA threshold effect value was set to 4, and the *p* value after FDR correction was set to 0.05, so as to find the flora markers with statistical differences between the two groups. It was found that there were 16 significantly different microorganisms in the P group, ranking from high to low according to LDA value were *c_Bacilli, o_Lactobacillales, p_Actinobacteria, f_Streptococcaceae, g_Streptococcus, c_Actinobacteria, o_Bifidobacteriales, f_Bifidobacteriaceae, g_Bifidobacterium, f_Lactobacillaceae, g_Lactobacillus, f_Enterobacteriaceae, o_Enterobacteriales, c_Gammaproteobacteria, f_Enterococcaceae,* and *g_Enterococcus*, while there were 11 significantly microbes in N group: *p_Bacteroidetes, c_Bacteroidia, o_Bacteroidales, f_Bacteroidaceae, g_Bacteroides, c_Clostridia, o_Clostridiales, f_Lachnospiraceae, g_Roseburia, g_Faecalibacterium,* and *f_Veillonellaceae*. Note: here p, c, o, f, g and s represent phylum, class, order, family, genus and species, respectively (Figures 2D,E).

**Figure 3:**
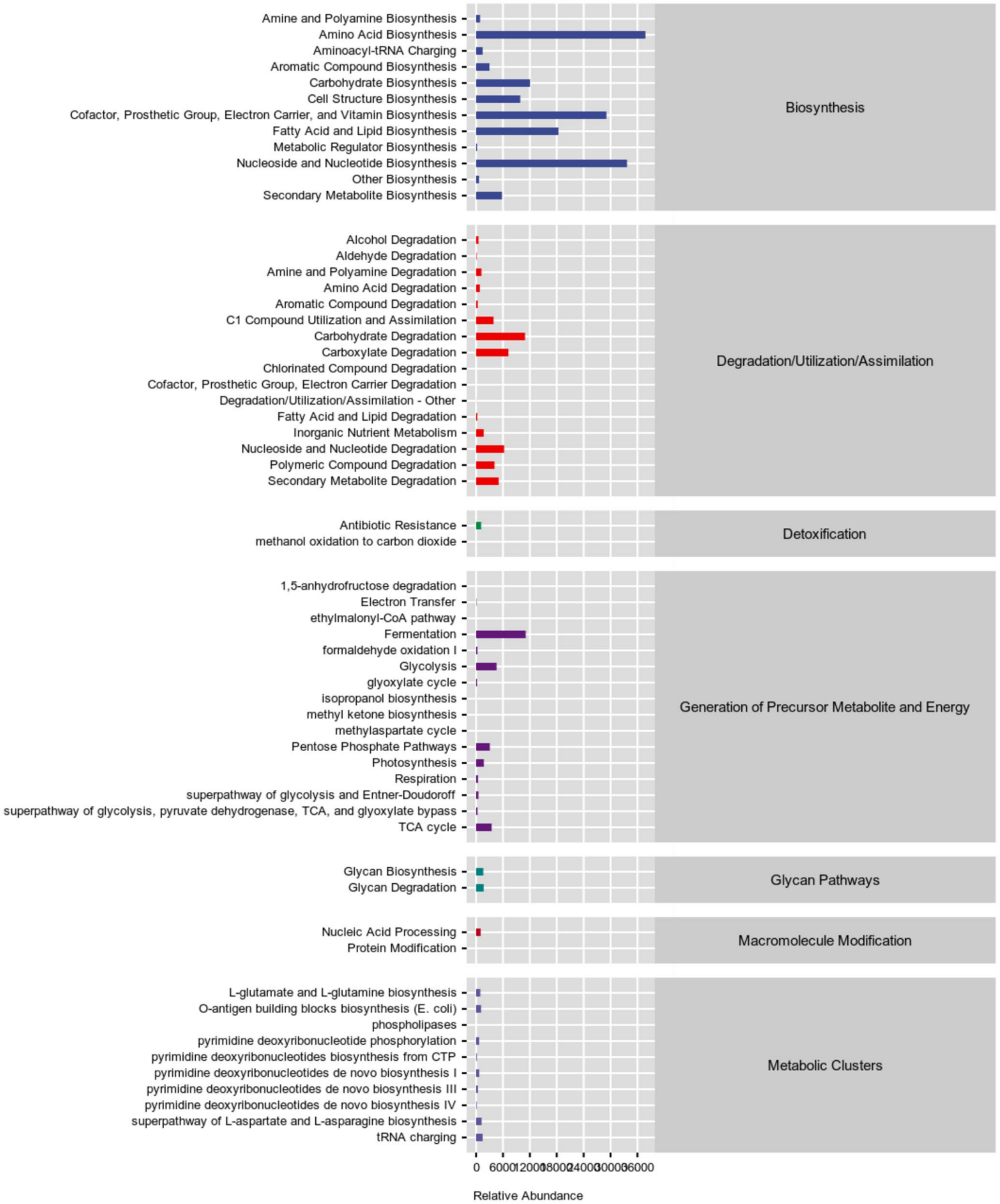
Statistics of metabolic pathways in PC patients. Different colors are used to distinguish different pathways, as blue represents biosynthensis, red represents degradation/utilization/assimilation, green represents detoxification, deep-purple represents generation of precursor metabolite and energy, blue-green represents glycan pathways, brown represents macromolecule modification, purple represents metabolic clusters.

Next, the 16S rRNA sequencing data were predicted by the phylogenetic investigation of communities by reconstruction of unobserved states (PICRUSt2) software. We used Kyoto Encyclopedia of Genes and Genomes (KEGG) pathway database^3^ and MetaCyc database^4^ annotated the functional path. Figure 3 and Supplementary Tables S1, S2 showed that the gut microbes of PC patients were clustered into six classifications, including metabolism, genetic information processing, environmental information processing, cellular processes, organismal systems, and human diseases in KEGG functional pathway analysis, while seven classifications in MetaCyc functional pathway analysis, among them, the carbohydrate metabolism pathway and amino acid biosynthesis pathway were significantly enriched, indicated that metabolic disorder may be related to GC metastasis. At the same time, 28 metabolic pathways were significantly different between P and N groups, the top five pathways ranked by difference from high to low were: mycothiol biosynthesis, mono-trans, poly-cis decaprenyl phosphate biosynthesis, methyl ketone biosynthesis, reductive acetyl coenzyme A pathway and superpathway of L-arginine, putrescine, and 4-aminobutanoate degradation (Table 2).

**Table 2:** Differential metabolic pathway between P and N groups (*P* < 0.05).

Pathway	Description	LogFC	SE	*P* value	adj *P* value
PWY1G-0	Mycothiol biosynthesis	2.615	0.321	4.44E-16	9.42E-14
PWY-6383	Mono-trans, poly-cis decaprenyl phosphate biosynthesis	2.316	0.284	4.44E-16	9.42E-14
PWY-7007	Methyl ketone biosynthesis	2.315	0.350	3.79E-11	5.35E-09
CODH-PWY	Reductive acetyl coenzyme A pathway	−2.359	0.371	2.05E-10	2.17E-08
ARGDEG-PWY	Superpathway of L-arginine, putrescine, and 4-aminobutanoate degradation	1.772	0.388	4.887E-06	<0.001
ORNARGDEG-PWY	Superpathway of L-arginine and L-ornithine degradation	1.772	0.388	4.887E-06	<0.001
PWY-6071	Superpathway of phenylethylamine degradation	1.763	0.380	3.561E-06	<0.001
PWY-5265	Peptidoglycan biosynthesis II (staphylococci)	1.821	0.408	7.96E-06	<0.001
3-HYDROXYPHENYLACETATE-DEGRADATION-PWY	4-hydroxyphenylacetate degradation	1.522	0.360	<0.001	0.001
PWY-6565	Superpathway of polyamine biosynthesis III	0.145	0.035	<0.001	0.002
PWY0-321	Phenylacetate degradation I (aerobic)	1.458	0.364	<0.001	0.002
PWY-5181	Toluene degradation III (aerobic) (*via* p-cresol)	1.466	0.369	<0.001	0.003
PWY0-1277	3-phenylpropanoate and 3-(3-hydroxyphenyl)propanoate degradation	1.566	0.405	<0.001	0.004
HCAMHPDEG-PWY	3-phenylpropanoate and 3-(3-hydroxyphenyl)propanoate degradation to 2-oxopent-4-enoate	1.725	0.451	<0.001	0.004
PWY-6690	Cinnamate and 3-hydroxycinnamate degradation to 2-oxopent-4-enoate	1.725	0.451	<0.001	0.004
GALLATE-DEGRADATION-II-PWY	Gallate degradation I	1.570	0.444	<0.001	0.011
PWY-6562	Norspermidine biosynthesis	1.416	0.409	0.001	0.014
GALLATE-DEGRADATION-I-PWY	Gallate degradation II	1.376	0.404	0.001	0.015
METHYLGALLATE-DEGRADATION-PWY	Methylgallate degradation	1.374	0.404	0.001	0.015
PWY-6185	4-methylcatechol degradation (ortho cleavage)	1.457	0.430	0.001	0.015
PWY-7373	Superpathway of demethylmenaquinol-6 biosynthesis II	2.106	0.667	0.002	0.032
ORNDEG-PWY	Superpathway of ornithine degradation	0.758	0.241	0.002	0.032
PWY-6397	Mycolyl-arabinogalactan-peptidoglycan complex biosynthesis	1.927	0.626	0.002	0.038
PWY-6182	Superpathway of salicylate degradation	1.260	0.411	0.002	0.038
PWY-5417	Catechol degradation III (ortho-cleavage pathway)	1.262	0.417	0.002	0.040
PWY-5431	Aromatic compounds degradation *via* &beta;-ketoadipate	1.262	0.417	0.002	0.040
CATECHOL-ORTHO-CLEAVAGE-PWY	Catechol degradation to &beta;-ketoadipate	1.285	0.426	0.003	0.040
VALDEG-PWY	L-valine degradation I	−1.354	0.453	0.003	0.042

#### *Streptococcus* is a gut microbial marker for predicting PC and PCLM

The top 20 important species were screened out by random forest (RF) analysis. The heat map showed the abundance distribution of these species in each sample (Figure 2F). Table 3 lists the top five ASV IDs and corresponding species, including *Streptococcus1, Streptococcus2, Streptococcus3, Streptococcus4, and Streptococcus5*. All of them were enriched in P group by LEfSe analysis, further analysis of PC prediction ability of the above species by ROC curve showed that the area under the curves (AUC) of *Streptococcus1* was 0.927 (*p* < 0.001), indicated that the increase of its number can predict the occurrence of PC (Figure 2G). In other words, *Streptococcus* is a predictive microbiota marker of PC, the more *Streptococcus* is, the higher the incidence of pancreatic cancer.

**Table 3:** Random forest model predicts the biomarkers for PC diagnosis.

Order	ASV	Bacteria	AUC	SE	*P*	Enriched group by LEfSe
1	ASV-130672	*Streptococcus1*	0.927	0.028	<0.001	P
2	ASV-46476	*Streptococcus2*	0.918	0.034	<0.001	P
3	ASV-80973	*Streptococcus3*	0.886	0.039	<0.001	P
4	ASV-48117	*Streptococcus4*	0.912	0.034	<0.001	P
5	ASV-75617	*Streptococcus5*	0.802	0.049	<0.001	P

To further explore the effect of fecal bacteria on PC patients with liver metastasis (LM), we divided them into liver metastasis group (LM group, *n* = 27) and non-liver metastasis group (non-LM group, *n* = 17), respectively. There was no significant difference in clinicopathological features between the two groups (Table 4). The sequencing depth of these two groups is good. The species composition of LM group is different from that of non-LM group, and the gene number and richness in LM group were higher. RF analysis found that *Streptococcus* also played a key role in identifying LM, and ROC curve was verified (AUC = 0.796, *p* = 0.001) (Supplementary Figures S1, S2 and Table 3), indicating that *Streptococcus* has great potential in predicting PC as well as PCLM.

**Table 4:** Demographic characteristics of LM (*n* = 27) and non-LM (*n* = 17) patients.

Variable	LM (*n* = 27)	non-LM (*n* = 17)	χ^2^/t	*P*
Age (y)				
<65	16 (59.26%)	11 (64.71%)	0.131	0.718
≥65	11 (40.74%)	6 (35.29%)		
Gender				
Male	14 (51.85%)	7 (41.18%)	0.477	0.490
Female	13 (48.15%)	10 (58.82%)		
BMI (kg/m^2^)	22.55 ± 3.10	22.23 ± 2.14	0.408	0.685
Tumor sites				
Head	9 (33.33%)	9 (52.94%)	1.931	0.587
Body	2 (7.41%)	1 (5.88%)		
Tail	3 (11.11%)	2 (11.76%)		
Whole	13 (48.15%)	5 (29.41%)		
Pathological types				
Adenocarcinoma	21 (77.78%)	15 (88.24%)	0.979	0.613
Squamous cell carcinoma	2 (7.41%)	1 (5.88%)		
Other	4 (14.81%)	1 (5.88%)		
Whether metastasis				
Yes	27 (100.00%)	5 (29.41%)	/	/
No	0 (0.00%)	12 (70.59%)		
Metastasis sites				
Liver	27 (100.00%)	0 (0.00%)	/	/
Lung	2 (7.41%)	2 (11.76%)		
Distant lymph node	15 (55.56%)	3 (17.65%)		
Peritoneum	7 (25.93%)	2 (11.76%)		
ECOG performance				
0	26 (96.30%)	0 (0.00%)	/	/
1	1 (3.70%)	17 (100.00%)		
Whether surgery			/	/
Yes	2 (7.41%)	9 (52.94%)		
No	25 (92.59%)	8 (47.06%)		
Total bilirubin (TBIL) (μmol/L)	16.04 ± 8.15	15.67 ± 17.69	0.088	0.931
Direct bilirubin (DBIL) (μmol/L)	6.12 ± 5.16	4.87 ± 4.33	0.771	0.446
Indirect bilirubin (IBIL) (μmol/L)	12.12 ± 9.79	11.47 ± 15.96	0.157	0.876

## Discussion

With the proposal of the Human Microbiome Project (HMP) and the extensive development of high-throughput sequencing, metagenomics, biochip technology and biological information analysis, the relationship between gut microbes and health has received unprecedented attention. Intestinal flora is an indispensable part of the body and plays an important role in human physiological health. It also plays a key role in the occurrence and development of pancreatic cancer (PC) (Riquelme et al., 2019). PC is one of the most lethal malignant tumors and one of the biggest burdens in the world. Because its early symptoms are not obvious and most of them are found in late stage, it is necessary to find convenient and non-invasive biomarkers. Therefore, in order to find microorganisms that may predict PC, we retrospectively collected fecal samples from 44 PC patients and 50 normal people, 16S rRNA sequencing technology and bioinformatic analysis were performed to find the predictive biomarkers.

Our study found that the intestinal microbial richness of PC patients was higher, and the *Streptococcus* content was significantly increased. Through LEfSe, RF analysis and verified by ROC curve, it was found that it had important discrimination ability in the PC group and could specifically predict PC and PCLM. *Streptococcus* belongs to *p__Firmicutes, c__Bacilli, o__Lactobacillales, and f__Streptococcaceae,* is a common pyogenic Gram-positive coccus, which widely exists in human gastrointestinal tract and nasopharynx, mainly causing pyogenic inflammation, hypersensitivity diseases and so on. Nowadays, its role in cancer occurrence and progression is gradually known (Boleij et al., 2011; Zhou et al., 2022). In the previous research of our team, we found that *Streptococcus* played a crucial role in GC and GCLM (Yu et al., 2021), and observed its role in extrahepatic metastasis of liver cancer in unpublished study. Thus, *Streptococcus* can be used as a biomarker for early diagnosis to guide the precise treatment of diseases. What’s more, in our preliminary functional and metabolic pathway analysis, we found that mycothiol biosynthesis pathway was significantly different between PC patients and normal people, and its changes may be a potential mechanism for the occurrence and development of PC. Mycothiol (MSH), a major low molecular weight thiol in mycobacteria, is an important cellular antioxidant. At present, a large number of literatures have reported that MSH is a promising antimicrobial target. As MSH only exists in actinomycetes, it is a good microbial target. However, as this study only explores PC and PCLM preliminarily, there is no in-depth mechanism exploration, and further *in vitro* and *in vivo* experiments are needed to prove it.

In addition, there are some limitations in our study: (1) this study is limited to a single center with a small sample size, which needs to be external verified by large sample and multi-center trials; (2) we could not avoid the impact of diet on intestinal microorganisms, we hope that randomized controlled trials can be conducted in the future to eliminate uncontrollable factors; (3) as the patients are still in the treatment stage and have not reached the follow-up time, we have not analyzed the treatment-related information in this study. Our research group has established a complete specimen bank of intestinal microbes of PC patients before and after treatment. We will conduct in-depth research in the future, hoping to reveal the potential synergy between tumor treatment and microbial microbes; (4) our study can only annotate microbial species at species levels, further *in vivo* animal experiments and clinical studies are needed to confirm the specific pathogens or bacteria that cause the differences; and (5) this study does not include the exploration of mechanism, we are looking forward to further exploration of mechanism by scholars based on our research findings in the future. Despite the above defects, this study can still explain the role of *Streptococcus* in PC and PCLM to a great extent.

## Conclusion

To sum up, the intestinal microbial structure characteristics of PC and PCLM patients have changed, and the number of *Streptococcus* in these two groups has increased significantly, which can specifically predict PC and PCLM and serve as a predictive microbiota marker.

## Data availability statement

The data presented in the study can be found in online repositories. The names of the repository and accession number can be found below: NCBI SRA, PRJNA977486, https://www.ncbi.nlm.nih.gov/sra/.

## Ethics statement

The studies involving human participants were reviewed and approved by the Union Hospital, Tongji Medical College, Huazhong University of Science and Technology. The patients/participants provided their written informed consent to participate in this study.

## Author contributions

JL and HL: conception and design and administrative support. YM: provision of study materials or patients. JY: collection and assembly of data. JY and YM: data analysis and interpretation. All authors contributed to the article and approved the submitted version.

## Funding

This work was supported by National Natural Science Foundation of China (No. 82103385), Chinese Society of Clinical Oncology Research Foundation (No. Y-tongshu2021/ms-0107), Beijing Bethune Public Welfare Foundation (No. BJ-GYQZHX2021006), and Hubei Xiaoping Chen Science and Technology Development Foundation Jingrui Development Fund (No. CXPJJH122006-1003).

## Conflict of interest

The authors declare that the research was conducted in the absence of any commercial or financial relationships that could be construed as a potential conflict of interest.

## Publisher’s note

All claims expressed in this article are solely those of the authors and do not necessarily represent those of their affiliated organizations, or those of the publisher, the editors and the reviewers. Any product that may be evaluated in this article, or claim that may be made by its manufacturer, is not guaranteed or endorsed by the publisher.

## Footnotes

^1^http://greengenes.secondgenome.com/

^2^https://view.qiime2.cn/

^3^http://www.genome.jp/kegg/pathway.html

^4^https://metacyc.org/

## Supplementary material

SUPPLEMENTARY FIGURE S1 16s rRNA sequencing data processing and species composition of LM and non-LM groups. **(A)** refraction curve; **(B)** species accumulation curve; **(C)** Venn gram of ASV/OTUs; **(D)** phylogenetic tree plot; **(E−H)** compositional analysis of each sample **(E,G)** and group **(F,H)** at the phylum level **(E,F)**, and at the genus level **(G,H)**.

SUPPLEMENTARY FIGURE S2 Species diversity results of LM and non-LM groups. **(A)** alpha diversity; **(B)** PCoA of beta diversity (R^2^=1.123, P=0.229); **(C)** 3D-PCoA; **(D)** taxonomic branch diagram of LEfSe (LDA threshold=2); **(E)** LDA histogram; **(F)** RF model; **(G)** ROC curves

## Abbreviations

PC: pancreatic cancer
LM: liver metastasis
rRNA: Ribosome ribonucleic acid
QIIME: Quantitative Insights into Microbial Ecology
ASVs: Amplicon sequence variants
PERMANOVA: permutational multivariate analysis of variance
PCoA: Principal coordinate analysis
LDA: linear discriminant analysis
LEfSe: linear discriminant analysis effect size
KEGG: Kyoto Encyclopedia of Genes and Genomes
