# Supplementary material for: Gut Streptococcus is a microbial marker for the occurrence and liver metastasis of pancreatic cancer
Source: Front Microbiol. 2023 Jun 14;14:1184869. doi: 10.3389/fmicb.2023.1184869 (PMC10306441; doi:10.3389/fmicb.2023.1184869)
Supplement: Supplementary file 1 [file Table_1.docx]

**Supplementary Table 1. KEGG functional pathway analysis in P and N groups**

| **Classifications** | **Functional pathways** | **Relative abundance** |
| --- | --- | --- |
| Metabolism  (n=11, average=2547.78) | Carbohydrate metabolism | 5410.53 |
|  | Metabolism of cofactors and vitamins | 4347.06 |
|  | Amino acid metabolism | 4333.38 |
|  | Metabolism of terpenoids and polyketides | 3258.40 |
|  | Metabolism of other amino acids | 2445.78 |
|  | Lipid metabolism | 2161.19 |
|  | Energy metabolism | 1971.73 |
|  | Glycan biosynthesis and metabolism | 1654.28 |
|  | Xenobiotics biodegradation and metabolism | 978.69 |
|  | Biosynthesis of other secondary metabolites | 750.35 |
|  | Nucleotide metabolism | 714.15 |
| Genetic Information Processing  (n=4, average=1163.18) | Replication and repair | 2073.06 |
|  | Folding, sorting and degradation | 1097.17 |
|  | Translation | 1127.90 |
|  | Transcription | 354.58 |
| Environmental Information Processing  (n=2, average=464.13) | Membrane transport | 783.69 |
|  | Signal transduction | 144.57 |
| Cellular Processes  (n=4, average=307.04) | Cell motility | 545.73 |
|  | Cell growth and death | 523.18 |
|  | Transport and catabolism | 96.76 |
|  | Cellular community – prokaryotes | 62.50 |
| Organismal Systems  (n=4, average=35.69) | Environmental adaptation | 69.68 |
|  | Endocrine system | 33.32 |
|  | Immune system | 23.53 |
|  | Digestive system | 16.23 |
| Human Diseases  (n=4, average=24.33) | Infectious diseases | 96.60 |
|  | Neurodegenerative diseases | 0.50 |
|  | Cardiovascular diseases | 0.19 |
|  | Immune diseases | 0.01 |

**Supplementary Table 2. MetaCyc functional pathway analysis in P and N groups**

| **Classifications** | **Functional pathways** | **Relative abundance** |
| --- | --- | --- |
| Biosynthesis  (n=12, average=12731.56) | Amino Acid Biosynthesis | 37785.24 |
|  | Nucleoside and Nucleotide Biosynthesis | 33678.67 |
|  | Cofactor, Prosthetic Group, Electron Carrier, and Vitamin Biosynthesis | 29084.49 |
|  | Fatty Acid and Lipid Biosynthesis | 18364.64 |
|  | Carbohydrate Biosynthesis | 12028.76 |
|  | Cell Structure Biosynthesis | 9852.25 |
|  | Secondary Metabolite Biosynthesis | 5774.68 |
|  | Aromatic Compound Biosynthesis | 2974.93 |
|  | Aminoacyl-tRNA Charging | 1460.03 |
|  | Amine and Polyamine Biosynthesis | 895.51 |
|  | Other Biosynthesis | 653.35 |
|  | Metabolic Regulator Biosynthesis | 226.11 |
| Degradation/ Utilization/ Assimilation  (n=15, average=2648.90) | Carbohydrate Degradation | 10911.55 |
|  | Carboxylate Degradation | 7208.91 |
|  | Nucleoside and Nucleotide Degradation | 6253.86 |
|  | Secondary Metabolite Degradation | 5033.83 |
|  | Polymeric Compound Degradation | 4100.87 |
|  | C1 Compound Utilization and Assimilation | 3885.19 |
|  | Inorganic Nutrient Metabolism | 1695.36 |
|  | Amine and Polyamine Degradation | 1188.45 |
|  | Amino Acid Degradation | 828.26 |
|  | Alcohol Degradation | 529.3 |
|  | Aromatic Compound Degradation | 364.77 |
|  | Fatty Acid and Lipid Degradation | 256.4 |
|  | Aldehyde Degradation | 104.29 |
|  | Degradation/Utilization/Assimilation - Other | 21.32 |
|  | Chlorinated Compound Degradation | 0.08 |
|  | Cofactor, Prosthetic Group, Electron Carrier Degradation | 0.01 |
| Glycan Pathways  (n=2, average=1650.37) | Glycan Degradation | 1694.06 |
|  | Glycan Biosynthesis | 1606.68 |
| Generation of Precursor Metabolite and Energy  (n=16, average=1610.90) | Fermentation | 11079.65 |
|  | Glycolysis | 4567.26 |
|  | TCA cycle | 3464.23 |
|  | Pentose Phosphate Pathways | 3062.54 |
|  | Photosynthesis | 1726.49 |
|  | superpathway of glycolysis and Entner-Doudoroff | 525.49 |
|  | Respiration | 437.28 |
|  | superpathway of glycolysis, pyruvate dehydrogenase, TCA, and glyoxylate bypass | 308.34 |
|  | formaldehyde oxidation I | 292.26 |
|  | glyoxylate cycle | 207.07 |
|  | Electron Transfer | 79.16 |
|  | isopropanol biosynthesis | 14.29 |
|  | methyl ketone biosynthesis | 5.39 |
|  | methylaspartate cycle | 3.47 |
|  | ethylmalonyl-CoA pathway | 1.14 |
|  | 1,5-anhydrofructose degradation | 0.38 |
| Metabolic Clusters  (n=10, average=680.37) | tRNA charging | 1460.03 |
|  | superpathway of L-aspartate and L-asparagine biosynthesis | 1220.57 |
|  | O-antigen building blocks biosynthesis (E. coli) | 1104.85 |
|  | L-glutamate and L-glutamine biosynthesis | 941.98 |
|  | pyrimidine deoxyribonucleotides de novo biosynthesis I | 692.76 |
|  | pyrimidine deoxyribonucleotide phosphorylation | 679.28 |
|  | pyrimidine deoxyribonucleotides de novo biosynthesis III | 413.43 |
|  | pyrimidine deoxyribonucleotides biosynthesis from CTP | 158.7 |
|  | pyrimidine deoxyribonucleotides de novo biosynthesis IV | 132.02 |
|  | phospholipases | 0.07 |
| Detoxification  (n=2, average=563.61) | Antibiotic Resistance | 1126.82 |
|  | methanol oxidation to carbon dioxide | 0.39 |
| Macromolecule Modification  (n=2, average=504.30) | Nucleic Acid Processing | 1008.57 |
|  | Protein Modification | 0.03 |

**Supplementary Table 3. Random Forest model predicts the biomarkers for PCLM diagnosis**

| **Order** | **ASV** | **Bacteria** | **AUC** | **SE** | ***P*** |
| --- | --- | --- | --- | --- | --- |
| 1 | ASV-45077 | *Bifidobacterium* | 0.362 | 0.094 | 0.126 |
| 2 | ASV-75617 | *Streptococcus6* | 0.663 | 0.093 | 0.071 |
| 3 | ASV-61341 | *Streptococcus7* | 0.796 | 0.071 | 0.001 |
| 4 | ASV-46476 | *Streptococcus8* | 0.646 | 0.094 | 0.106 |
| 5 | ASV-106607 | *Streptococcus9* | 0.692 | 0.083 | 0.034 |
