# Supplementary figures and images for: Gut Streptococcus is a microbial marker for the occurrence and liver metastasis of pancreatic cancer
Source: Front Microbiol. 2023 Jun 14;14:1184869. doi: 10.3389/fmicb.2023.1184869 (PMC10306441; doi:10.3389/fmicb.2023.1184869)

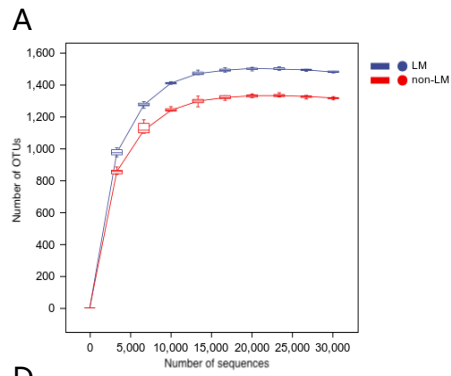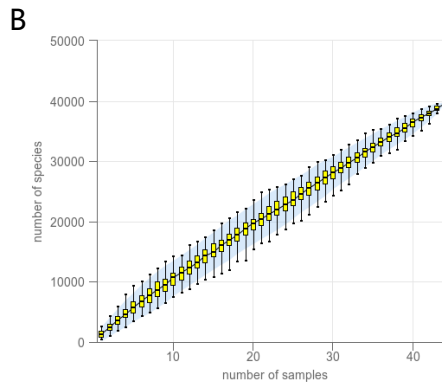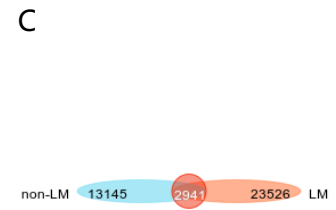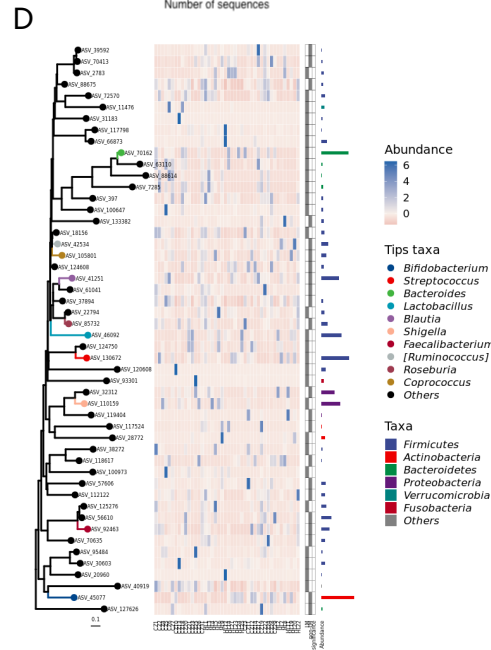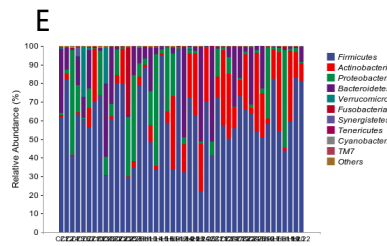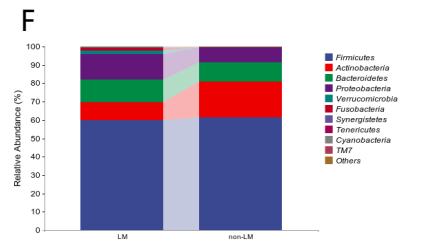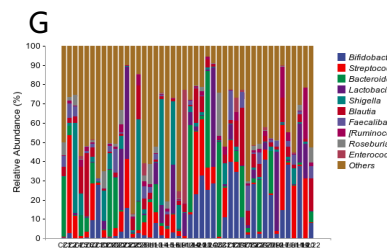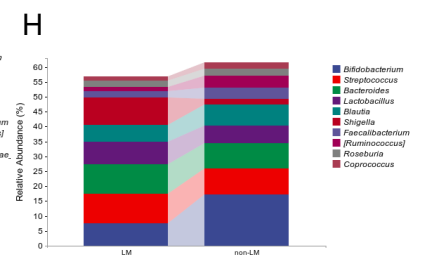

Supplement: SUPPLEMENTARY FIGURE S1 — 16s rRNA sequencing data processing and species composition of LM and non-LM groups. (A) refraction curve; (B) species accumulation curve; (C) Venn gram of ASV/OTUs; (D) phylogenetic tree plot; (E−H) compositional analysis of each sample (E,G) and group (F,H) at the phylum level (E,F), and at the genus level (G,H). [file Image_1.pdf]

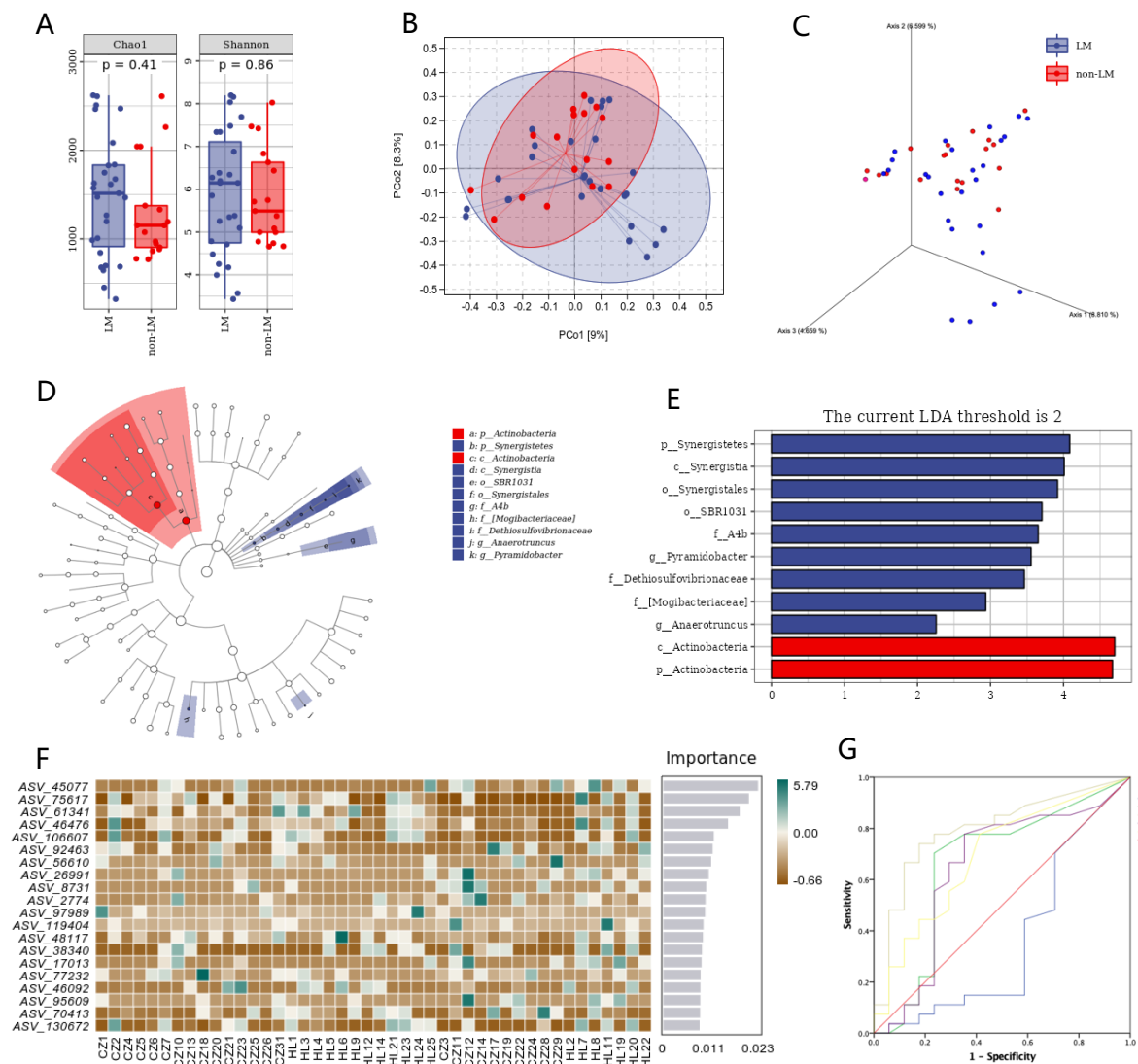

Supplement: SUPPLEMENTARY FIGURE S2 — Species diversity results of LM and non-LM groups. (A) alpha diversity; (B) PCoA of beta diversity (R2=1.123, P=0.229); (C) 3D-PCoA; (D) taxonomic branch diagram of LEfSe (LDA threshold=2); (E) LDA histogram; (F) RF model; (G) ROC curves [file Image_2.pdf]
